# Dyspepsia and Vinegar

**Published:** 1854-11

**Authors:** 


DYSPEPSIA AND VINEGAR.—As soon as food reaches the stomach of a hungry, healthy man, it pours out a fluid substance, called *gastric juice*, as instantly as the eye yields water, if it is touched by any thing hard; this gastric dissolves the food from without inwards, as lumps of ice in a glass of water are melted from without inwards. If from any cause the food is not thus melted, or dissolved, that is *indigestion*, or *dyspepsia*. Vinegar, in its action on food, is more nearly like the gastric juice than any other fluid known; thus it is that a pickle or a little vinegar will "*settle the stomach*," when some discomfort is experienced after eating. The best vinegar for that or any other purpose is made thus: Mix two quarts of New Orleans molasses with five quarts of warm rain water, and four quarts of yeast: use in a few weeks.

